# Distinct prognostic trajectories of heart failure phenotypes following kidney transplantation

**DOI:** 10.1093/eschf/xvag221

**Published:** 2026-08-20

**Authors:** Maor Bril, Ashraf Imam, Elchanan Parnasa, Michael Rivin, Suha Shabaneh, Keren Tzukert, Abel Roai, Offer Amir, Abed Khalaileh, Rabea Asleh

**Affiliations:** Heart Institute, Hadassah Medical Center, Faculty of Medicine, Hebrew University of Jerusalem, P.O.B 12000, Jerusalem 91112001, Israel; Department of General Surgery and Organ Transplantation Unit, Hadassah Medical Center, Faculty of Medicine, Hebrew University of Jerusalem, P.O.B 12000, Jerusalem 91112001, Israel; Heart Institute, Hadassah Medical Center, Faculty of Medicine, Hebrew University of Jerusalem, P.O.B 12000, Jerusalem 91112001, Israel; Department of General Surgery and Organ Transplantation Unit, Hadassah Medical Center, Faculty of Medicine, Hebrew University of Jerusalem, P.O.B 12000, Jerusalem 91112001, Israel; Department of General Surgery and Organ Transplantation Unit, Hadassah Medical Center, Faculty of Medicine, Hebrew University of Jerusalem, P.O.B 12000, Jerusalem 91112001, Israel; Department of Nephrology, Hadassah Medical Center, Faculty of Medicine, Hebrew University of Jerusalem, P.O.B 12000, Jerusalem 91112001, Israel; Department of Nephrology, Hadassah Medical Center, Faculty of Medicine, Hebrew University of Jerusalem, P.O.B 12000, Jerusalem 91112001, Israel; Heart Institute, Hadassah Medical Center, Faculty of Medicine, Hebrew University of Jerusalem, P.O.B 12000, Jerusalem 91112001, Israel; Department of General Surgery and Organ Transplantation Unit, Hadassah Medical Center, Faculty of Medicine, Hebrew University of Jerusalem, P.O.B 12000, Jerusalem 91112001, Israel; Heart Institute, Hadassah Medical Center, Faculty of Medicine, Hebrew University of Jerusalem, P.O.B 12000, Jerusalem 91112001, Israel

**Keywords:** Heart failure, HFpEF, HFrEF, Kidney transplantation, Uraemic cardiomyopathy, Cardiac remodelling

## Abstract

**Background and Aims:**

Heart failure (HF) drives post-kidney transplant (KT) morbidity. While KT reverses components of uraemic cardiomyopathy, the persistence of post-transplant risk in HF with preserved (HFpEF) versus HF with reduced ejection fraction (HFrEF) remains unclear. We evaluated phenotype-specific remodelling and clinical outcomes.

**Methods:**

Retrospective cohort of adult KT recipients stratified by pre-transplant echocardiography: HFpEF (left ventricular ejection fraction [LVEF] ≥50% with elevated filling pressures), HFrEF (LVEF <50%), or controls. Longitudinal echocardiographic remodelling and post-transplant outcomes were analysed using multivariable Cox regression.

**Results:**

Of 442 recipients, 64 (14.5%) had HFpEF, 44 (10.0%) HFrEF, and 334 (75.5%) controls. Over a 49-month median follow-up, HFpEF was independently associated with HF hospitalization (adjusted hazard ratio [aHR] 9.57; 95% confidence interval [CI] 3.37–27.2, *P* < .001) and composite death/HF hospitalization (aHR 4.46; 95% CI 2.36–8.42, *P* < .001). Paired longitudinal echocardiography demonstrated persistent diastolic stiffness within the HFpEF group (E/e': 12.7 ± 4.6 to 11.8 ± 5.3, *P* = .117). Conversely, HFrEF patients exhibited systolic recovery (ΔLVEF +8.7 ± 10.2%, *P* = .003), remaining independently associated with all-cause mortality (aHR 2.9; 95% CI 1.16–7.25, *P* = .023) but not HF hospitalization (aHR 3.11; 95% CI 0.71–13.6, *P* = .13). Mechanistically, ΔLVEF predicted lower HF hospitalization risk (aHR 0.94; 95% CI 0.90–0.99, *P* = .02), whereas mortality tracked with continuous changes in haemoglobin (aHR 0.71; 95% CI 0.60–0.83, *P* < .001) and calcium (aHR 0.69; 95% CI 0.50–0.96, *P* = .027).

**Conclusions:**

Post-KT trajectories differ by pre-transplant HF phenotype. Uraemic HFpEF is a persistent, non-reversible phenotype driving severe post-KT morbidity. HFrEF demonstrates systolic reversibility. Pre-transplant evaluation should incorporate targeted diastolic profiling beyond standard LVEF-centred assessment to identify KT candidates at high risk for recurrent post-transplant HF morbidity.

## Graphical Abstract

**Graphical Abstract:**
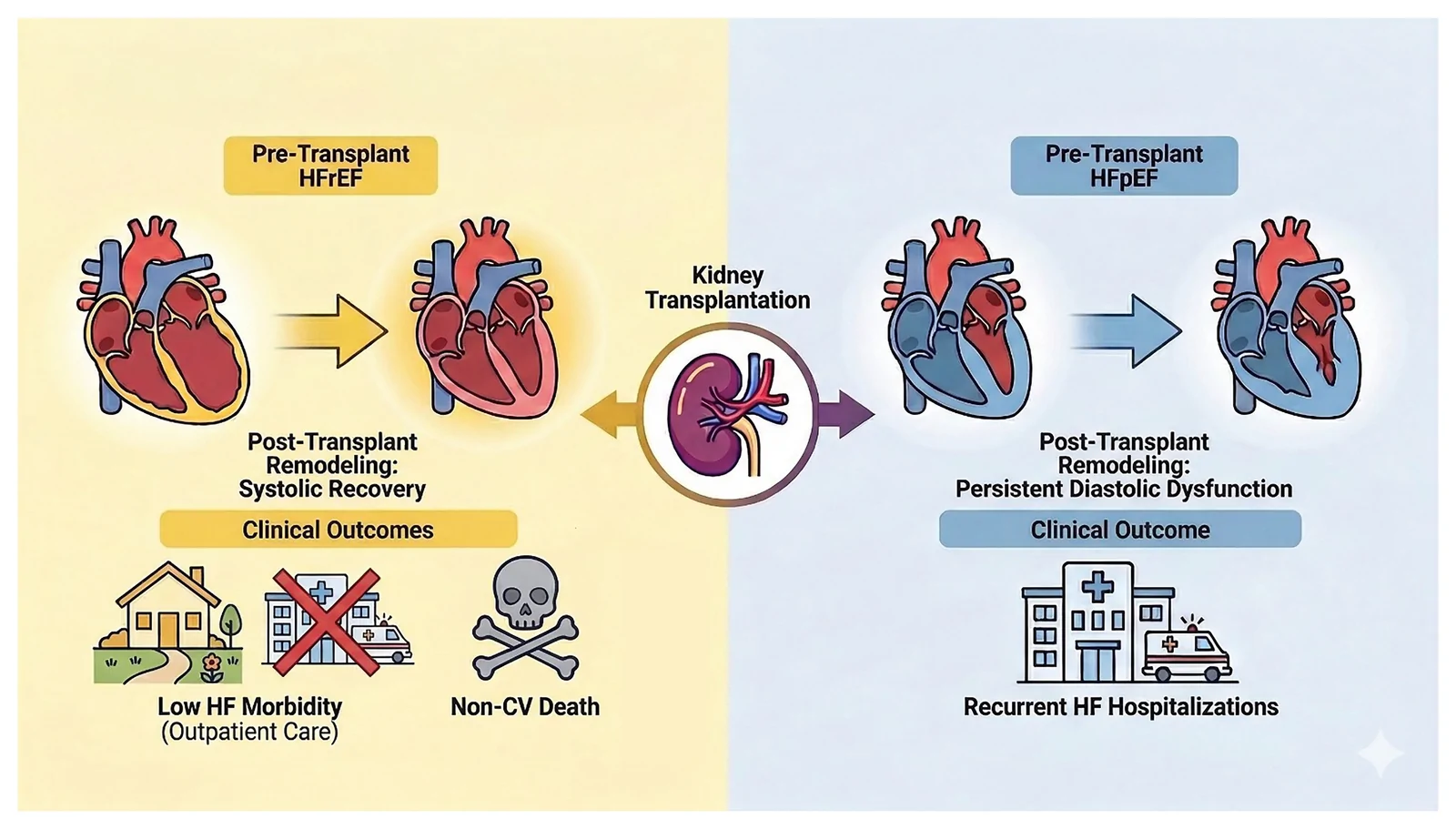
Divergent Cardiac Trajectories of HFrEF and HFpEF After Kidney Transplantation. This illustration highlights the contrasting clinical pathways of patients with pre-existing HFrEF and those with HFpEF following kidney transplantation. While patients with HFrEF predominantly experience myocardial reverse remodelling and low rates of HF recurrence, they remain at risk for non-cardiovascular mortality. In contrast, those with HFpEF exhibit persistent diastolic dysfunction, translating to a high burden of recurrent HF hospitalizations.

## Introduction

Cardiovascular disease remains the leading cause of death after kidney transplant (KT), accounting for a significant burden of late mortality and graft loss despite successful restoration of renal function.^1–3^ Although KT provides a substantial survival advantage compared with maintenance dialysis, cardiovascular risk is incompletely mitigated, reflecting pathophysiologic mechanisms that extend beyond traditional risk factors.^2,3^ In end-stage kidney disease (ESKD), chronic exposure to uraemia promotes a distinct myocardial phenotype, commonly termed uraemic cardiomyopathy, which is characterized by left ventricular hypertrophy, diffuse interstitial fibrosis, microvascular dysfunction, and abnormalities of both diastolic and systolic performance.^4–7^ This remodelling syndrome arises from a complex interaction of haemodynamic factors, especially persistent pressure and volume overload, anaemia, and arteriovenous shunting, alongside non-haemodynamic factors, including disrupted calcium-phosphate metabolism, oxidative stress, inflammation, and retention of cardiotoxic uraemic solutes.^4,5,8^ Consequently, myocardial injury in ESKD often represents structural and metabolic remodelling driven by the uraemic milieu rather than purely ischaemic damage.

Importantly, the myocardial substrate differs substantially between heart failure with reduced ejection fraction (HFrEF) and heart failure with preserved ejection fraction (HFpEF) in this population. In HFrEF, ventricular dilation and impaired contractility may partially reverse following restoration of kidney function and reduction in afterload and neurohormonal stress. Observational studies have demonstrated meaningful improvement in left ventricular ejection fraction (LVEF) after KT, even among patients with ischaemic heart disease.^9–11^ Accordingly, contemporary pre-transplant cardiovascular assessment has traditionally emphasized the detection of obstructive coronary artery disease and overt systolic dysfunction.^12^ In contrast, uraemic HFpEF is typically characterized by concentric remodelling, extracellular matrix expansion, increased myocardial stiffness, impaired relaxation, and reduced diastolic reserve.^4,6,8,13^ These structural abnormalities reflect diffuse interstitial fibrosis and microvascular dysfunction, hallmarks of HFpEF pathobiology, conditions that may not be reversible after KT.^13,14^ Unlike the volume-responsive dilation observed in HFrEF, the fibrotic extracellular matrix expansion underlying HFpEF may persist despite normalization of renal function, thereby maintaining elevated filling pressures and limiting haemodynamic adaptability. Whether KT meaningfully alters this fibrotic trajectory remains uncertain.

Diagnostic and risk stratification of HFpEF in ESKD present additional challenges. Dialysis-associated volume shifts confound symptom-based functional classification, while structural criteria, such as left ventricular hypertrophy, lack specificity given their high prevalence in advanced kidney disease.^4,15^ Registry-based analyses frequently rely on administrative coding rather than granular echocardiographic characterization, obscuring phenotype-specific risk.^2,3,16^ Furthermore, although guidelines strongly recommend screening for ischaemia and systolic dysfunction prior to KT, they provide limited guidance on assessing diastolic function, myocardial stiffness, or diastolic reserve.^12^ Advanced echocardiographic techniques, including global longitudinal strain (GLS), detect subclinical dysfunction and independently predict cardiovascular events and mortality in KT recipients, suggesting that an LVEF-centric paradigm may underestimate risk in patients with preserved ejection fraction.^17–19^ Collectively, these gaps underscore the need to define whether HFrEF and HFpEF follow divergent remodelling and prognostic trajectories after KT. Clarifying phenotype-specific responses to renal restoration has direct implications for perioperative risk stratification and post-transplant management.

In this study, we sought to characterize the longitudinal cardiac remodelling patterns and clinical outcomes of KT recipients with pre-existing HFrEF and HFpEF in a large contemporary cohort. Using comprehensive echocardiographic profiling, we aimed to (i) delineate phenotype-specific remodelling trajectories following KT; (ii) compare risks of mortality, cardiovascular morbidity, and graft loss; and (iii) identify structural and metabolic predictors associated with adverse outcomes.

## Methods

### Study design and population

We conducted a single-centre retrospective cohort study of consecutive adult patients who underwent KT at Hadassah University Medical Center between January 2014 and May 2024. Clinical, laboratory, transplant-related, and echocardiographic data were collected directly from the institutional electronic medical records. Eligible participants were adults (≥18 years) with an available pre-transplant transthoracic echocardiogram adequate for assessment of left ventricular systolic and diastolic function. To ensure evaluation of post-transplant cardiovascular trajectory, patients were required to have a functioning graft beyond the immediate post-operative period. Individuals with peri-operative mortality, primary non-function, or early graft loss due to surgical or immunologic causes were excluded. Follow-up extended from the date of KT until death, graft failure, last clinical contact, or study censoring in August 2025. The investigation conforms with the principles outlined in the Declaration of Helsinki. The study protocol was approved by the ethics committee at Hadassah University Medical Center. Given its retrospective design, the requirement for written informed consent was waived.

### Definition of heart failure (HF) phenotypes

Patients were classified according to pre-transplant echocardiographic phenotype. HFrEF was defined as an LVEF <50%. HFpEF was defined as LVEF ≥50% in conjunction with objective evidence of elevated filling pressures. HFpEF was defined using a pre-specified hierarchical algorithm that required either a lateral E/e′ ratio >13 or a lateral E/e′ ratio between 9 and 13 in combination with left atrial enlargement defined as a left atrial volume index ≥34 ml/m^2^ or sex-specific enlargement by linear dimension (left atrial diameter ≥4.0 cm in men and ≥3.8 cm in women). A documented clinical diagnosis of HFpEF was considered supportive but not sufficient in isolation in sensitivity analyses. The lateral E/e′ threshold was selected in accordance with the American Society of Echocardiography (ASE) and the European Association of Cardiovascular Imaging (EACVI) recommendations, recognizing higher tissue Doppler velocities at the lateral annulus compared with septal measurements.^20,21^ Patients with preserved LVEF who did not meet echocardiographic or clinical criteria for HF comprised the control group.

### Clinical variables and transplant characteristics

Baseline variables were collected at the time of KT and included demographics, comorbidities, laboratory parameters, and transplant-related characteristics. Dialysis vintage was defined as the cumulative duration of dialysis exposure prior to KT; patients undergoing pre-emptive transplantation were assigned a vintage of zero. Delayed graft function was defined as the requirement for dialysis within the first week after surgery. Comorbid conditions, including hypertension, diabetes mellitus, ischaemic heart disease, and peripheral vascular disease, were confirmed through active diagnostic coding and physician documentation. Cardiovascular interventions occurring before or after KT were systematically adjudicated through detailed chart review and categorized as ischaemic revascularization procedures, electrophysiologic interventions (including ablation or device implantation), and valvular interventions (including surgical or transcatheter valve repair or replacement). Laboratory parameters used for longitudinal analyses were obtained from a standardized post-transplant surveillance window between 9 and 12 months after KT to minimize perioperative confounding and capture stabilized graft function. Longitudinal laboratory analyses focused on haemoglobin, calcium, and phosphate because these parameters were systematically available across the cohort within the standardized post-transplant surveillance window and reflect correction of key uraemic-metabolic disturbances after KT. Natriuretic peptides and the urinary albumin-to-creatinine ratio were not routinely measured across the full cohort during the study period. Serum creatinine and eGFR were available as part of routine transplant follow-up and were used clinically for graft-failure ascertainment; however, formal longitudinal eGFR trajectory modelling was not included in the present cardiac phenotype-focused analysis.

### Echocardiographic assessment

Transthoracic echocardiography was performed according to contemporary ASE/EACVI recommendations. LVEF was quantified primarily using the modified Simpson biplane method with three-dimensional volumetric assessment incorporated when image quality permitted.^21,22^ Linear or visual estimation methods were used only when endocardial delineation was suboptimal. Diastolic function assessment included lateral mitral annular tissue Doppler velocity (e′), E/e′ ratio, and left atrial size indexed to body surface area. Left ventricular internal diameter in diastole (LVIDd) and right ventricular systolic pressure (RVSP), estimated from tricuspid regurgitation velocity, were recorded as markers of chamber remodelling and pulmonary haemodynamics. The baseline echocardiogram corresponded to the most recent examination prior to transplantation. Post-transplant echocardiography was performed based on clinical indication or targeted surveillance of high-risk individuals, reflecting real-world practice. Reverse remodelling was defined as the absolute change (*Δ*) between post-transplant and baseline measurements.

### Clinical endpoints

The primary outcome was a composite of all-cause mortality or hospitalization for HF. Secondary outcomes included the individual outcomes of all-cause mortality, first hospitalization for HF, and death-censored graft failure defined as return to chronic dialysis or re-transplantation. In patients with pre-existing HFrEF or HFpEF, HF hospitalization represented decompensation of established disease, while in the control group, hospitalization for HF represented incident HF.

## Statistical analysis

Continuous variables are reported as mean ± standard deviation (SD) if normally distributed or as median with interquartile range (IQR) if not normally distributed. Categorical variables are presented as counts and percentages. Baseline characteristics were compared using one-way analysis of variance (ANOVA) or the Kruskal–Wallis test for continuous variables and the Chi-square or Fisher's exact tests for categorical variables.

Longitudinal changes in echocardiographic parameters were evaluated using paired statistical testing, and between-group differences in remodelling were assessed using covariance models adjusted for baseline values to mitigate regression-to-the-mean bias. Survival curves were generated using the Kaplan–Meier method and compared using the log-rank test. Multivariable Cox proportional hazards regression was used to estimate hazard ratios (HR) and 95% confidence intervals (CI) for clinical outcomes. A hierarchical adjustment strategy was prespecified, incorporating demographic variables, cardiovascular comorbidities, and dialysis vintage as a surrogate for cumulative uraemic exposure and adverse outcomes after KT.^23^ The proportional hazards assumption was evaluated using Schoenfeld residuals. Missing data were handled using complete-case analysis; patients with missing values for specific covariates were excluded from the respective Cox models.

For the analysis of graft failure, standard Cox models treated death as a censoring event. Additionally, because mortality may inherently preclude the occurrence of graft loss, a competing-risk regression using the Fine–Gray method was performed, treating death with a functioning graft as a competing event. Sensitivity analyses excluded patients classified as HFpEF solely on the basis of administrative coding. Additional models examined the prognostic significance of longitudinal changes in cardiac structure, mineral metabolism parameters, and haemoglobin levels. No formal correction for multiple comparisons was applied. The primary endpoint and the main phenotype-based Cox models were prespecified, whereas secondary endpoints, longitudinal echocardiographic comparisons, and exploratory analyses of metabolic predictors should be considered hypothesis-generating. All analyses were performed using R version 4.4.1 (R Foundation for Statistical Computing). A two-sided *P*-value <.05 was considered statistically significant.

## Results

### Study population

**Figure 1:**
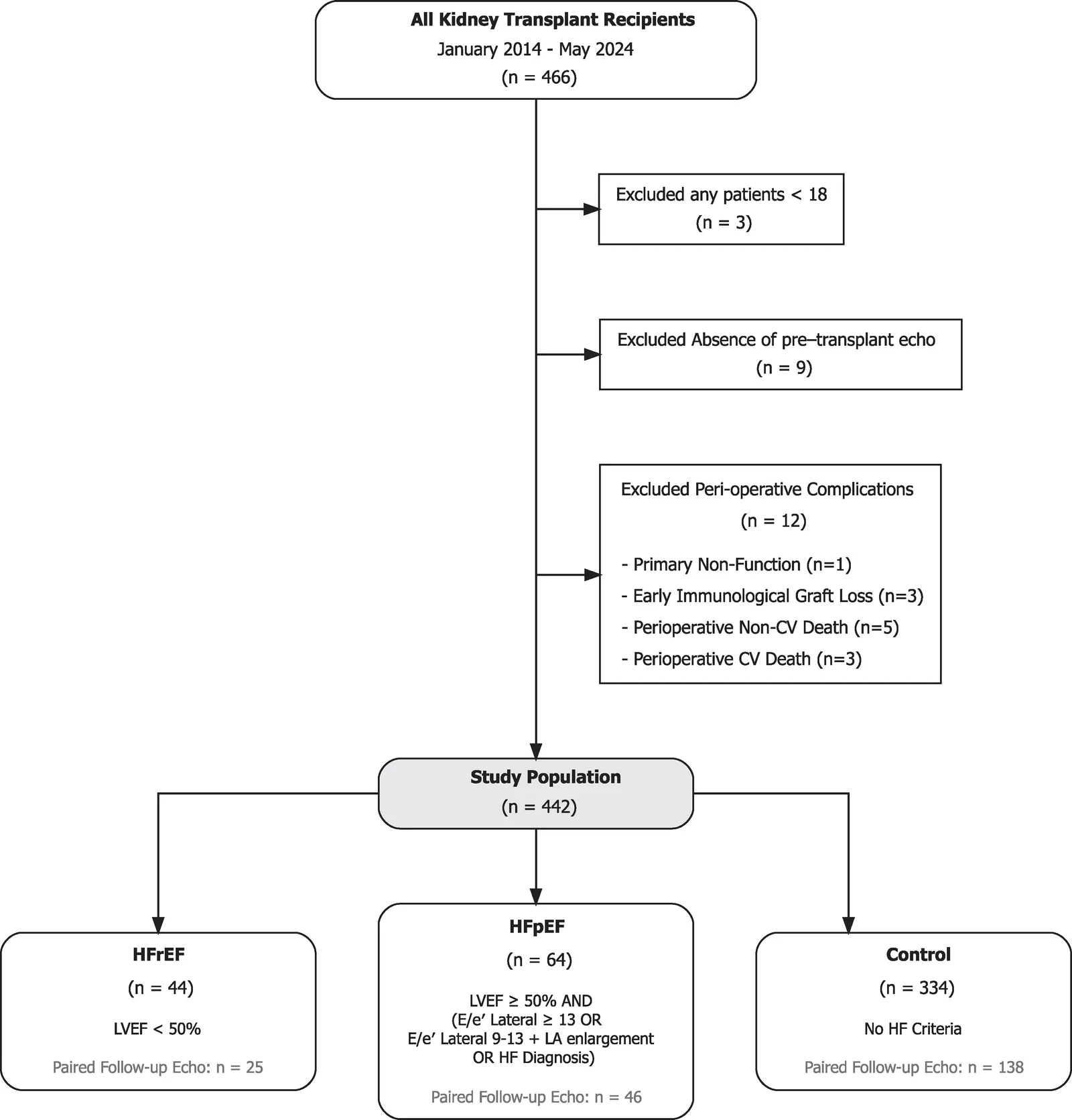
Study population inclusion and exclusion flowchart. Kidney transplant recipients between January 2014 and May 2024. Patients were excluded based on age (<18 years), absence of pre-transplant echocardiography, or severe perioperative complications (including technical failure, primary non-function, early immunological graft loss, and perioperative death). The final study population (*n* = 442) was stratified into three groups based on pre-transplant baseline echocardiographic and clinical criteria: heart failure with reduced ejection fraction (HFrEF, *n* = 44), heart failure with preserved ejection fraction (HFpEF, *n* = 64), and controls without heart failure (HF) (*n* = 334). The number of recipients with available paired pre- and post-transplant echocardiography is shown for each phenotype group and serves as the denominator for longitudinal remodelling analyses.

Between January 2014 and May 2024, 466 KT were performed at our centre. After excluding paediatric recipients (*n* = 3), patients without adequate pre-transplant echocardiography (*n* = 9), and cases of perioperative mortality or early graft loss (*n* = 12), the final analysis included 442 adult KT recipients (*Figure 1*). Based on pre-transplant echocardiographic phenotype, 44 patients (10%) were classified as HFrEF, 64 (14.5%) as HFpEF, and 334 (77.5%) as controls without HF.

Baseline characteristics differed substantially across phenotypes (*Table 1*). Recipients with HF were older (mean age ± SD: HFrEF, 57.0 ± 12.7 years; HFpEF, 55.8 ± 14.2 years) than controls (50.2 ± 14.0 years) and exhibited a greater burden of cardiometabolic comorbidity. Diabetes and hypertensive nephropathy were the predominant causes of ESKD in both HF groups, whereas the control group had more heterogeneous aetiologies, including genetic and immunologic causes. Dialysis exposure differed markedly across groups. Median dialysis vintage was longest in HFrEF (40 months), intermediate in HFpEF (27.5 months), and shortest in controls (15 months), with pre-emptive KT most frequent in controls (16.5%). The HFrEF group was predominantly male (90.9%) and had the highest prevalence of ischaemic heart disease (61.4%). The HFpEF group demonstrated a cardiometabolic risk profile comparable to HFrEF, with high rates of diabetes and hypertension, the highest body mass index (27.9 ± 5.1 kg/m^2^), and a greater proportion of women (32.8%) relative to HFrEF.

**Table 1:** Baseline patient characteristics

Characteristics	HFrEF (*n* = 44)	HFpEF (*n* = 64)	Control (*n* = 334)	*P*-value
**Demographics**				
Age (years)	57.0 ± 12.7	55.8 ± 14.2	50.2 ± 14.0	<.001
Male sex	40 (90.9%)	43 (67.2%)	242 (72.5%)	.015
BMI (kg/m^2^)	26.1 ± 4.5	27.9 ± 5.1	27.0 ± 4.8	.198
**Aetiology of end-stage kidney disease** ^a^				
Diabetes mellitus	16 (36.4%)	24 (37.5%)	51 (15.3%)	<.001
Hypertension	14 (31.8%)	21 (32.8%)	49 (14.7%)	<.001
IGA nephropathy	2 (4.5%)	3 (4.7%)	37 (11.1%)	.139
Polycystic disease	5 (11.4%)	0 (0.0%)	44 (13.2%)	.009
Urologic cause	4 (9.1%)	3 (4.7%)	20 (6.0%)	.633
Other/unknown	12 (27.3%)	24 (37.5%)	159 (47.6%)	.020
**Transplant details**				
Transplant number				.615
First	41 (93.2%)	56 (87.5%)	296 (88.6%)	
Re-transplant (2+)	3 (6.8%)	8 (12.5%)	38 (11.4%)	
Dialysis modality				.084
Haemodialysis	35 (83.3%)	49 (77.8%)	230 (70.3%)	
Peritoneal dialysis	5 (11.9%)	10 (15.9%)	43 (13.1%)	
Pre-emptive transplant	2 (4.8%)	4 (6.3%)	54 (16.5%)	
Time on dialysis (months)				<.001
Median (*Q*1, *Q*3)	40.0 (14.5, 62.0)	27.5 (12.5, 61.5)	15.0 (4.0, 42.0)	
Donor type				.017
Deceased donor (DD)	9 (20.5%)	24 (37.5%)	63 (19.0%)	
Donation after circulatory death (DCD)	1 (2.3%)	0 (0.0%)	3 (0.9%)	
Living donor (LD)	34 (77.3%)	40 (62.5%)	265 (80.1%)	
Delayed graft function (DGF)	6 (13.6%)	11 (17.2%)	24 (7.2%)	.024
**Comorbidities**				
Diabetes mellitus	18 (40.9%)	31 (48.4%)	73 (21.9%)	<.001
Hypertension	39 (88.6%)	59 (92.2%)	279 (83.5%)	.162
Ischemic heart disease	27 (61.4%)	11 (17.2%)	38 (11.4%)	<.001
Peripheral vascular disease	4 (9.1%)	6 (9.4%)	17 (5.1%)	.290

Values are mean ± SD, median (IQR), or *n* (%).

*P*-values: One-way ANOVA for normally distributed variables, Kruskal–Wallis test for skewed variables, and Chi-square test for categorical variables.

BMI, body mass index; HFpEF, heart failure with preserved ejection fraction; HFrEF, heart failure with reduced ejection fraction.

^a^Some patients had combined diabetic and hypertensive nephropathy.

### Echocardiographic reverse remodelling

**Figure 2:**
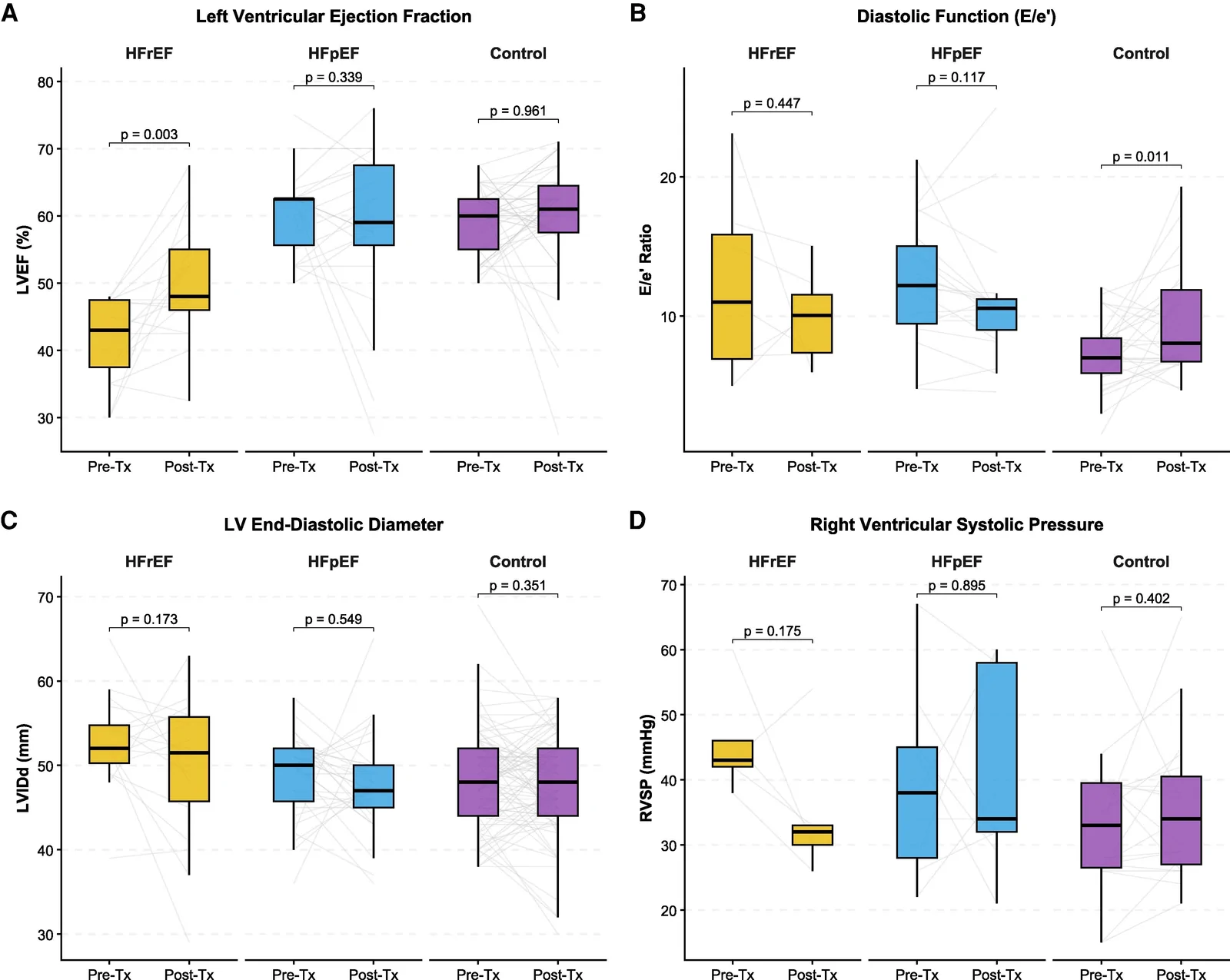
Longitudinal changes in echocardiographic parameters after kidney transplantation. Paired analysis of echocardiographic parameters before (pre-Tx) and after (post-Tx) kidney transplantation across the three study groups (HFrEF, HFpEF, and Control). (*A*) Left ventricular ejection fraction (LVEF, %). (*B*) Diastolic function assessed by E/e ratio. (*C*) Left ventricular end-diastolic diameter (LVIDd, mm). (*D*) Right ventricular systolic pressure (RVSP, mmHg). Boxplots represent the median and interquartile range (IQR), with lines connecting individual patient trajectories to demonstrate paired changes. *P*-values denote the statistical significance of within-group changes following kidney transplantation.

Paired pre- and post-transplant echocardiographic data were available for analysis in 209 KT recipients. Longitudinal remodelling trajectories are shown in *Figure 2*, and absolute changes (*Δ*) across phenotypes are detailed in *Table 2*. Recipients with HFrEF demonstrated significant systolic recovery following KT. Among those with available paired measurements (*n* = 86), mean LVEF improved from 42.2 ± 5.9% pre-transplant to 51.5 ± 8.7% post-transplant (mean ΔLVEF +8.7%, *P* = .003). Although reductions in LV dimensions (ΔLVIDd −3.0 mm) and RVSP (ΔRVSP −10.8 mmHg) were observed, these changes did not reach statistical significance, likely reflecting heterogeneity and the modest sample size of specific paired subsets (*n* = 33 for ΔRVSP). These findings are consistent with a reverse remodelling pattern characterized primarily by systolic functional recovery. In contrast, HFpEF recipients exhibited limited improvement in filling pressures. Within the paired tissue Doppler subset (*n* = 55), mean E/e′ decreased modestly from 12.7 to 11.8, but this change did not reach statistical significance (*P* = .117). Post-transplant filling pressures remained elevated relative to controls, suggesting persistent diastolic stiffness despite renal recovery. Notably, the control group demonstrated a modest but statistically significant increase in E/e′ during follow-up (*Δ* +2.1, *P* = .011). While mean imaging intervals were longer in the control group (30.8 ± 26.4 months), covariate adjustment confirmed that scan intervals did not confound the overall between-group differences in diastolic remodelling.

**Table 2:** Echocardiographic reverse remodelling

Echo parameter	*N*	HFrEF (*n* = 44)	HFpEF (*n* = 64)	Control (*n* = 334)	*P*-value
Time from pre-KT echo to KT (months)	442	7.8 ± 7.3	12.4 ± 12.1	12.8 ± 10.7	<.001
Time from KT to follow-up echo (months)	209	19.7 ± 16.4	17.7 ± 21.4	30.8 ± 26.4	.001
LVEF (%)—baseline	276	42.2 ± 5.9	60.7 ± 5.6	60.0 ± 4.3	<.001
LVEF (%)—Follow-up	134	51.5 ± 8.7	59.2 ± 10.9	59.2 ± 8.2	.002
Δ LVEF (%)	86	8.7 ± 10.2	−2.5 ± 12.0	−0.1 ± 10.3	.006
E/e′ lateral—Baseline	206	9.5 ± 5.2	12.7 ± 4.6	7.0 ± 2.0	<.001
E/e’ lateral—Follow-up	103	9.6 ± 3.4	11.8 ± 5.3	9.6 ± 3.5	.2
Δ E/e′ lateral	55	−2.3 ± 7.4	−1.4 ± 3.7	2.1 ± 4.2	.023
LVIDd (mm)—Baseline	426	54.0 ± 5.8	49.0 ± 4.8	47.9 ± 5.2	<.001
LVIDd (mm)—Follow-up	130	49.9 ± 8.3	47.7 ± 5.7	47.6 ± 6.1	.5
*Δ* LVIDd (mm)	128	−3.0 ± 8.9	−0.9 ± 8.0	−0.7 ± 6.3	.6
RVSP (mmHg)—Baseline	154	38.9 ± 10.9	36.1 ± 11.9	30.7 ± 8.3	.004
RVSP (mmHg)—Follow-up	81	34.1 ± 8.6	38.4 ± 19.5	34.6 ± 13.8	.7
*Δ* RVSP (mmHg)	33	−10.8 ± 14.7	0.9 ± 19.6	2.4 ± 12.3	.3

Values are mean ± SD. *P*-values calculated using One-way ANOVA.

Baseline and follow-up columns include all available cross-sectional data at each time point. The *Δ* values and corresponding *P*-values represent longitudinal changes calculated exclusively within the paired subset.

*Δ*, follow-up minus baseline; HFpEF, heart Failure with preserved ejection fraction; HFrEF, heart failure with reduced ejection fraction; KT, kidney transplantation; LVEF, left ventricular ejection fraction; LVIDd, left ventricular internal diameter (diastole); RVSP, right ventricular systolic pressure.

Comparison of recipients with and without detailed follow-up echocardiography (Supplementary Table S1) confirmed that, within the HFrEF and HFpEF groups, baseline characteristics and clinical outcomes did not differ significantly by imaging availability, supporting the representativeness of remodelling analyses. In contrast, the control subgroup with longitudinal imaging had a higher baseline cardiovascular risk profile than controls without longitudinal imaging, including a greater prevalence of ischaemic heart disease and higher adverse event rates. Thus, the control comparator in our remodelling analyses was conservatively biased towards higher risk.

### Post-transplant clinical outcomes

**Figure 3:**
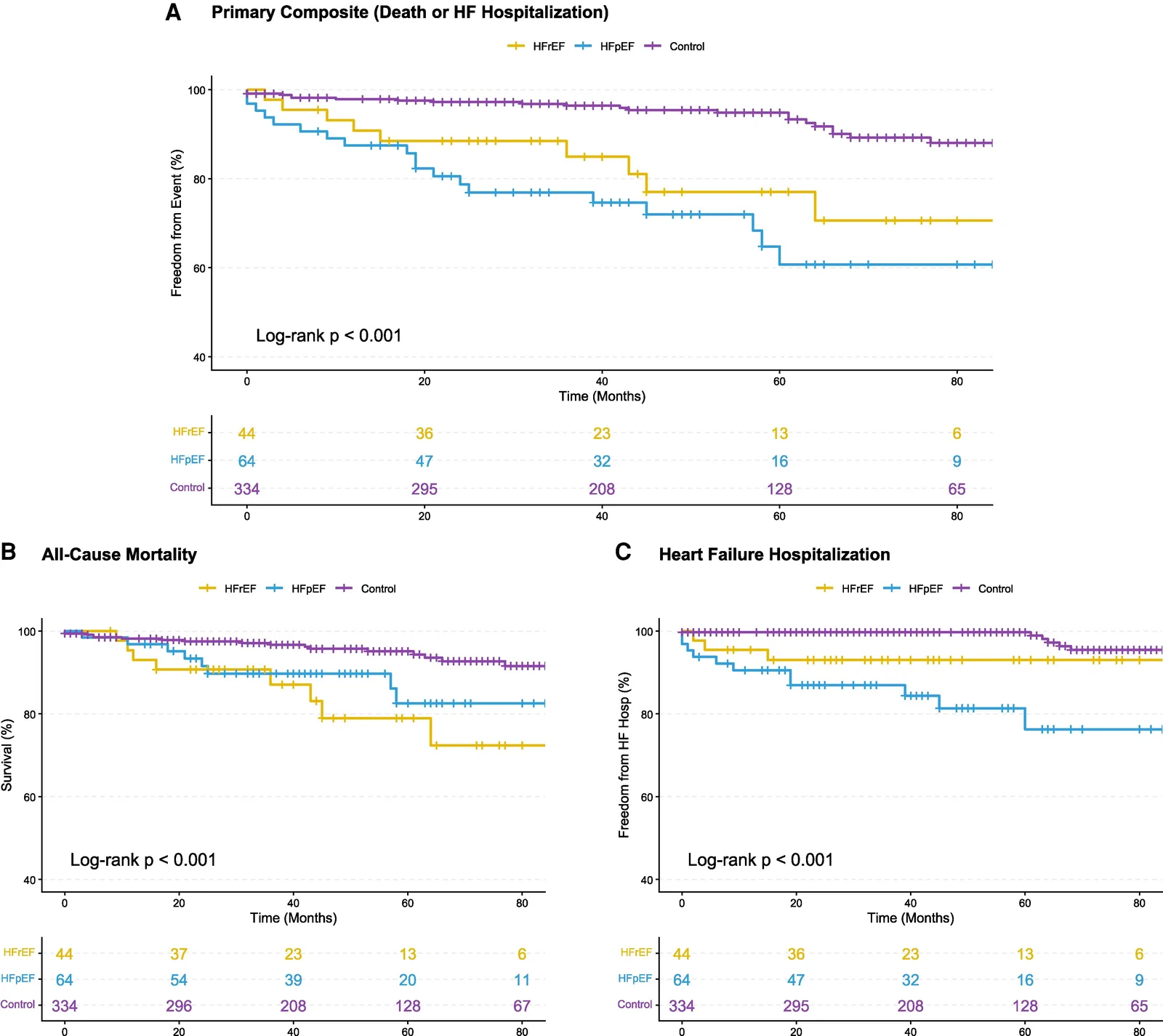
Kaplan-Meier estimates of post-transplant clinical outcomes among kidney transplant recipients stratified by their pre-transplant HF phenotype. (*A*) Freedom from the primary composite outcome, defined as the first occurrence of either all-cause mortality or HF hospitalization. (*B*) Freedom from all-cause mortality. (*C*) Freedom from the first HF hospitalization. The number at risk for each group at specified time intervals (in months) is provided below the plot. Statistical comparisons between the groups were performed using the overall log-rank test.

Over a median follow-up of 49 months (IQR 27–73), 35 deaths (7.9%) and 21 HF hospitalizations (4.8%) occurred. Event rates differed significantly by phenotype (Supplementary Table S2). Both HF phenotypes were associated with a substantially higher risk of the primary composite endpoint (death or HF hospitalization) compared with controls. In unadjusted analysis, HFpEF carried the highest risk (HR 5.95, 95% CI 3.24–10.9, *P* < .001), followed by HFrEF (HR 3.51, 95% CI 1.62–7.63, *P* = .002). After full multivariable adjustment, both HF phenotypes remained independently associated with the primary composite. HFpEF maintained the highest risk (adjusted HR 4.46, 95% CI 2.36–8.42, *P* < .001), and HFrEF also demonstrated a persistent independent association (adjusted HR 2.82, 95% CI 1.22–6.52, *P* = .016), indicating a significant prognostic burden across both groups (*Table 3*, *Figure 3A*).

**Table 3:** Association of HF phenotypes with clinical outcomes

Characteristic	Unadjusted			Adjusted^a^		
	HR	95% CI	*P*-value	HR	95% CI	*P*-value
Primary Composite Outcome (death or HF hospitalization)						
Control	—	—		—	—	
HFrEF	3.51	1.62, 7.63	**.002**	2.82	1.22, 6.52	**.016**
HFpEF	5.95	3.24, 10.9	**<.001**	4.46	2.36, 8.42	**<.001**
All-cause mortality						
Control	—	—		—	—	
HFrEF	3.76	1.63, 8.66	**.002**	2.90	1.16, 7.25	**.023**
HFpEF	2.92	1.31, 6.53	**.009**	1.68	0.73, 3.90	.2
HF hospitalization						
Control	—	—		—	—	
HFrEF	4.30	1.07, 17.2	**.039**	3.11	0.71, 13.6	.13
HFpEF	11.7	4.33, 31.9	**<.001**	9.57	3.37, 27.2	**<.001**
Death-censored graft loss						
Control	—	—		—	—	
HFrEF	3.06	0.82, 11.4	.095	2.39	0.60, 9.53	.2
HFpEF	4.32	1.50, 12.4	**.007**	2.60	0.88, 7.66	.084

Bold values indicate significance at *P* < 0.05.

CI, confidence interval; HFpEF, heart Failure with preserved ejection fraction; HFrEF, heart failure with reduced ejection fraction; HR, hazard ratio.

^a^Adjusted for age, sex, diabetes mellitus, ischaemic heart disease, and dialysis vintage.

Analysis of the secondary outcomes, including the individual components of the primary composite endpoint, demonstrated divergent prognostic trajectories for the two HF phenotypes. All-cause mortality was highest in HFrEF (*Figure 3B*). In adjusted models, HFrEF remained independently associated with mortality (adjusted HR, 2.9; 95% CI, 1.16–7.25; *P* = .023). Although crude mortality was elevated in HFpEF (14% vs 5.4% in controls), the association attenuated after adjustment (adjusted HR 1.68, 95% CI 0.73–3.90, *P* = .20), suggesting that excess mortality in HFpEF is largely influenced by the burden of associated comorbidities (*Table 3*).

In contrast, HF hospitalization was predominantly driven by HFpEF (*Figure 3C*). Incidence was 17% in HFpEF compared with 6.8% in HFrEF and 2.1% in controls (*P* < .001). After full adjustment, HFpEF remained strongly associated with the risk of hospitalization (adjusted HR 9.57, 95% CI 3.37–27.2, *P* < .001). The magnitude and stability of this association across hierarchical models in HFpEF (Supplementary Table S3) underscore the robustness of the phenotype-specific morbidity signal. Notably, HFrEF was not independently associated with HF hospitalization after adjustment (*P* = .13), consistent with the systolic recovery observed in this group.

Death-censored graft failure occurred in 18 recipients (4.1%). In standard unadjusted analysis, HFpEF was associated with a higher risk of graft loss (HR 4.32, 95% CI 1.5–12.4, *P* = .007). While this association attenuated to a borderline trend in the fully adjusted Cox model (adjusted HR 2.6, 95% CI 0.88–7.66, *P* = .084; *Table 3*), the risk remained robust when statistically accounting for death as a competing risk. In a Fine–Gray competing risk regression, HFpEF remained independently associated with a significantly higher sub-distribution hazard (sHR) for graft loss after full multivariable adjustment (adjusted sHR 2.36, 95% CI 1.04–5.33, *P* = .04; Supplementary Table S4). In contrast, no significant associations with graft failure were observed for the HFrEF phenotype or the control group in both standard Cox and competing-risk-adjusted models.

As expected, pre-transplant coronary revascularization was more prevalent in HFrEF (percutaneous coronary intervention [PCI] 41%, coronary artery bypass grafting [CABG] 16%) than in HFpEF (PCI 9.4%, CABG 7.8%), consistent with the higher prevalence of ischaemic cardiomyopathy in HFrEF. Post-transplant coronary event rates did not differ significantly among groups (Supplementary Table S2).

### Sensitivity and exploratory analyses

To validate the specificity of the HFpEF phenotype and to rule out potential confounding from misclassification or inconsistent approaches to defining the group, a sensitivity analysis was conducted. Restricting HFpEF classification to patients who met strict echocardiographic criteria and lacked a prior administrative clinical diagnosis (*n* = 46) yielded consistent findings. HFpEF remained independently associated with HF hospitalization (adjusted HR 7.39, 95% CI 2.31–23.7, *P* < .001), supporting the validity of the phenotype.

Finally, to explore the mechanisms driving mortality in the HFrEF group, an exploratory landmark survival analysis was performed using longitudinal echocardiographic data from HFrEF patients. Among HFrEF recipients with paired echocardiographic imaging (*n* = 16), an exploratory landmark analysis showed that patients achieving ≥10% absolute LVEF improvement (defined as responders) had no deaths during follow-up, whereas mortality occurred exclusively among non-responders. Although underpowered (*P* = .14), this signal supports the prognostic importance of systolic reverse remodelling.

### Prognostic value of longitudinal changes in cardiac and metabolic predictors

We further examined whether longitudinal changes (*Δ*) in echocardiographic and metabolic parameters could predict long-term outcomes. Longitudinal changes revealed a mechanistic dissociation between cardiac-specific recovery and systemic metabolic restoration (*Table 4*). Each 1% increase in ΔLVEF was associated with a 6% relative reduction in future HF hospitalization risk (adjusted HR 0.94, *P* = .02), identifying systolic recovery as the primary determinant of freedom from HF events. In contrast, overall survival and graft preservation were more strongly associated with correction of the uraemic-metabolic axis. Improvement in haemoglobin was the strongest independent predictor of survival (adjusted HR 0.71 per 1 g/dL increase, *P* < .001), alongside favourable changes in calcium and phosphate levels. These findings suggest that while cardiac reverse remodelling mitigates HF morbidity, long-term survival is predominantly influenced by restoration of renal-metabolic homeostasis.

**Table 4:** Prognostic value of longitudinal changes in cardiac and metabolic parameters

Characteristic	All-cause mortality			HF hospitalization			Graft failure		
	HR	95% CI	*P*-value	HR	95% CI	*P*-value	HR	95% CI	*P*-value
*Δ* LVEF (Per 1% improvement)	1.01	0.95, 1.09	.7	0.94	0.90, 0.99	**.020**	0.98	0.93, 1.04	.5
*Δ* E/e’ ratio (Per 1 unit change)	0.92	0.78, 1.10	.4	0.96	0.82, 1.12	.6	1.98	0.93, 4.19	.074
*Δ* Haemoglobin (Per 1 g/dl increase)	0.71	0.60, 0.83	**<.001**	0.91	0.73, 1.13	.4	0.70	0.56, 0.88	**.002**
*Δ* Calcium (Per 1 mg/dl increase)	0.69	0.50, 0.96	**.027**	1.19	0.80, 1.77	.4	0.54	0.37, 0.81	**.002**
*Δ* Phosphate (Per 1 mg/dl increase)	1.16	0.96, 1.40	.13	1.13	0.88, 1.44	.3	1.30	1.05, 1.60	**.016**

Bold values indicate significance at *P* < 0.05.

*Δ*, differences between last follow-up and baseline; CI, confidence interval, HR, hazard ratio; LVEF, left ventricular ejection fraction.

HR with 95% CI were derived from Cox proportional hazards regression models and adjusted for age and sex.

## Discussion

HF represents one of the most prevalent and prognostically significant complications in patients with advanced CKD and among KT candidates. Contemporary data demonstrate that HF, across both reduced and preserved ejection fraction phenotypes, is strongly associated with post-transplant mortality, graft dysfunction, and recurrent hospitalization, as well as a heavy burden of comorbid frailty which impairs quality of life.^1,16,24–26^ Although KT is frequently conceptualized as a therapy capable of reversing uraemic cardiomyopathy, accumulating evidence suggests that cardiac response after KT is heterogeneous.^1,5^ In this contemporary cohort of KT recipients stratified by pre-transplant HF subtype using objective echocardiographic criteria, we observed a clear divergence in post-transplant cardiovascular trajectories. While KT recipients with HFrEF demonstrated substantial systolic reverse remodelling accompanied by attenuation of HF-related morbidity, those with HFpEF exhibited persistent diastolic dysfunction and a marked, independent excess risk of HF hospitalization despite restoration of renal function. These findings suggest that the cardiovascular response to KT is phenotype-specific and that reliance on LVEF alone may underestimate residual post-transplant risk in patients with HFpEF.

A central finding of our study is the emergence of uraemic HFpEF as a persistent, non-reversible, and high-morbidity phenotype. Unlike the reversible nature of some uraemic cardiomyopathies, HFpEF exhibited limited evidence of diastolic improvement after KT and was associated with a substantially elevated risk of HF hospitalization. Although the relatively low absolute event count for post-transplant HF hospitalizations limits model precision, this phenotype-specific morbidity signal demonstrated marked stability across sequentially adjusted nested models (Supplementary Table S3) and strict sensitivity analyses. This consistency suggests that the excess risk reflects intrinsic myocardial pathology rather than transient volume fluctuations. These findings are consistent with contemporary HF literature emphasizing that HFpEF is characterized by concentric remodelling, interstitial fibrosis, microvascular dysfunction, and impaired diastolic reserve.^20,27^ In CKD populations, HFpEF has emerged as a dominant and prognostically important phenotype. Translational studies of CKD-related cardiomyopathy further highlight metabolic remodelling and fibrosis as central mechanisms that may not fully regress after correction of renal dysfunction.^4,5^

The absence of meaningful improvement in filling pressure indices in our HFpEF cohort supports the hypothesis that once diastolic stiffness is established, it may be only partially reversible. This may explain why some studies show modest improvement in conventional diastolic indices after kidney transplantation, whereas patients with more advanced or structurally established HFpEF continue to exhibit persistent diastolic abnormalities and recurrent congestion.^28^ Unlike the eccentric remodelling typical of HFrEF, the fibrosis-dominant substrate of HFpEF may lack sufficient structural plasticity to normalize compliance after KT. Persistent impairment of diastolic reserve likely explains the disproportionate burden of recurrent congestion and post-transplant HF morbidity observed in this phenotype.

In contrast, our findings reinforce the concept that a substantial component of left ventricular systolic dysfunction in patients with ESKD is potentially reversible following KT. Recipients with HFrEF showed meaningful systolic recovery after KT, with a mean LVEF improvement of nearly 9%, consistent with prior observational studies showing reverse cardiac remodelling after restoration of renal function.^9–11,24^ Several mechanisms likely contribute to this improvement. First, KT corrects the chronic volume and pressure overload characteristic of ESKD, thereby reducing ventricular wall stress and afterload mismatch and allowing recovery of myocardial contractile performance. Second, KT reverses key components of the uraemic milieu, including retention of uraemic toxins, oxidative stress, inflammation, and adverse metabolic signalling, all of which contribute to impaired myocardial energetics and excitation-contraction coupling in uraemic cardiomyopathy.^4^ Third, recovery of graft function often leads to correction of anaemia and improvement in oxygen delivery, which may reduce high-output stress and improve myocardial efficiency. In parallel, restoration of renal function has been associated with improvement in cardiovascular functional reserve, suggesting that improvement in cardiac performance extends beyond resting ejection fraction.^29^ Finally, more sensitive imaging markers indicate that myocardial recovery after KT extends beyond conventional LVEF. Contemporary strain imaging studies demonstrate improvement in global longitudinal strain and myocardial mechanics after KT, which correlates with better clinical outcomes and may represent an earlier marker of myocardial recovery than LVEF alone.^18^ Taken together, these mechanisms suggest that LVEF recovery after KT reflects the combined effects of haemodynamic unloading, metabolic restoration, and partial reversal of structural uraemic cardiomyopathy, supporting the concept that systolic dysfunction in many patients with advanced kidney disease represents a reversible cardiorenal phenotype.

However, despite systolic recovery, HFrEF remained associated with excess mortality compared with controls. This apparent dissociation between mechanical improvement and survival aligns with modern concepts of renal-cardiac crosstalk, which emphasize that CKD induces systemic metabolic, inflammatory, and vascular remodelling that may persist despite restoration of renal filtration.^4,5^ In this framework, KT may improve ventricular performance yet fail to fully reverse the systemic ‘legacy’ of long-standing uraemia. While our exploratory analysis hinted that mortality clustered among patients without systolic recovery, this subgroup was explicitly underpowered. Consequently, this observation is strictly hypothesis-generating, though it conceptually aligns with the notion that reversibility of myocardial dysfunction may be the key determinant of prognosis within HFrEF.

A key insight of our study is the identification of distinct determinants of HF morbidity and overall survival. Improvement in systolic mechanics (ΔLVEF) independently predicted freedom from HF hospitalization, underscoring the importance of reverse remodelling in mitigating the risk of HF hospitalization. In contrast, long-term survival and graft retention were more closely associated with systemic metabolic recovery, particularly correction of anaemia and mineral metabolism. These findings align with broader CKD literature demonstrating that outcomes in HF-CKD overlap syndromes are governed not only by cardiac structure but also by systemic metabolic and inflammatory processes.^4,5,30^ We propose a dual-axis model of post-transplant risk: a cardiac-mechanical axis influencing HF morbidity and a renal-metabolic axis determining survival and graft stability. Recognition of these parallel pathways may refine post-transplant surveillance and therapeutic prioritization.

Current pre-transplant cardiovascular assessment remains heavily focused on ischaemia detection and LVEF thresholds. However, contemporary AHA scientific statements have emphasized the need for more nuanced, risk-based approaches rather than routine ischaemia screening alone.^12,15^ Our data suggest that reliance on preserved LVEF may underestimate risk in patients with objective diastolic dysfunction. HFpEF recipients, despite preserved EF, experienced the highest burden of post-transplant HF hospitalization. These findings support incorporation of structured diastolic profiling, including objective filling pressure assessment, into KT evaluation algorithms. Although our current study is limited by the retrospective unavailability of advanced deformation imaging for baseline phenotyping, future incorporation of parameters such as GLS could further refine this evaluation, as GLS may identify subclinical myocardial disease not reflected in LVEF.^18^ A phenotype-driven approach could allow earlier identification of high-risk HFpEF recipients and enable pre-emptive post-transplant volume and blood pressure management, tailored follow-up, and consideration of targeted HFpEF therapies, such as non-steroidal mineralocorticoid receptor antagonists, which significantly reduce HF hospitalizations in cardiorenal patients with baseline hypertrophy.^31^

### Study limitations

This study has several limitations. First, its retrospective, single-centre design introduces inherent selection bias. KT candidates with advanced, decompensated HF are typically excluded from listing, resulting in a relatively stable HF cohort. Second, pre-transplant echocardiography was not standardized relative to dialysis sessions, which may introduce volume-related variability. Additionally, because the 10-year inclusion period witnessed sequential updates in ASE/EACVI diastolic guidelines, some temporal heterogeneity in imaging acquisition and reporting is inherent. Third, post-transplant echocardiographic surveillance was driven primarily by clinical indications rather than a strict, protocol-specified timeline. This non-randomized approach introduces selection bias in opposite directions: it conservatively shifts the control arm towards higher-risk individuals who required repeat evaluation, while potentially underestimating the true magnitude of myocardial recovery in HF cohorts by disproportionately capturing patients with persistent symptoms. Fourth, detailed longitudinal data on the optimization of guideline-directed medical therapy and on specific cardiovascular biomarkers, including natriuretic peptides, were unavailable. The urinary albumin-to-creatinine ratio was also not systematically measured across the full cohort. Although serum creatinine and eGFR were available as part of routine transplant follow-up, formal modelling of longitudinal eGFR trajectories, a recognized prognostic marker in HF,^32^ was beyond the scope of the present cardiac phenotype-focused analysis because post-transplant renal function may act as both a mediator and an outcome.

## Conclusions

In KT recipients, pre-transplant HF phenotype identifies distinct post-transplant cardiovascular trajectories. HFpEF represents a persistent, high-risk phenotype characterized by sustained diastolic dysfunction and disproportionate HF hospitalization despite restoration of renal function. In contrast, HFrEF frequently demonstrates systolic reversibility with reduced HF morbidity, although survival remains influenced by systemic comorbidity. These findings challenge an LVEF-centric paradigm and support a shift towards phenotype-based evaluation incorporating objective diastolic assessment and myocardial mechanics. Recognition of HFpEF before KT may enable more precise risk stratification and tailored post-transplant management strategies to reduce recurrent HF events.

## Supplementary Material

xvag221_Supplementary_Data

## Data Availability

The data underlying this article will be shared on reasonable request to the corresponding author. All authors declare no funding for this contribution. Ethical Approval was not required. None supplied.
