# Supplementary material for: Distinct prognostic trajectories of heart failure phenotypes following kidney transplantation
Source: ESC Heart Fail. 2026 Aug 20;13(5):xvag221. doi: 10.1093/eschf/xvag221 (PMC13531464; doi:10.1093/eschf/xvag221)
Supplement: Supplementary file 1 [file xvag221_supplementary_data.docx]

**Supplementary Materials**

**Supplementary Table S1.** Baseline Characteristics and Outcomes Stratified by Availability of Paired Echocardiographic Data before and after kidney transplantation for each phenotype.

|  | **HFrEF** | | | **HFpEF** | | | **Control** | | |
| --- | --- | --- | --- | --- | --- | --- | --- | --- | --- |
| **Characteristic** | **No / Limited Follow-up Echo**  N = 23 | **Detailed Follow-up Echo**  N = 21 | **p-value** | **No / Limited Follow-up Echo**  N = 31 | **Detailed Follow-up Echo**  N = 33 | **p-value** | **No / Limited Follow-up Echo**  N = 254 | **Detailed Follow-up Echo**  N = 80 | **p-value** |
| Age (Years) | 56 (13) | 58 (12) | 0.4 | 52 (15) | 59 (12) | 0.094 | 49 (14) | 53 (13) | **0.020** |
| Male Sex | 20 (87%) | 20 (95%) | 0.6 | 23 (74%) | 20 (61%) | 0.2 | 178 (70%) | 64 (80%) | 0.083 |
| BMI (kg/m²) | 26.9 (5.4) | 25.4 (3.4) | 0.3 | 27.0 (4.7) | 28.6 (5.4) | 0.2 | 27.1 (4.9) | 26.9 (4.6) | 0.8 |
| Diabetes Mellitus | 9 (39%) | 9 (43%) | 0.8 | 16 (52%) | 15 (45%) | 0.6 | 51 (20%) | 22 (28%) | 0.2 |
| Ischemic Heart Disease | 12 (52%) | 15 (71%) | 0.2 | 5 (16%) | 6 (18%) | 0.8 | 18 (7.1%) | 20 (25%) | **<0.001** |
| Dialysis Vintage (Months) | 45 (31) | 40 (40) | 0.3 | 42 (51) | 49 (50) | 0.5 | 27 (36) | 38 (53) | 0.14 |
| Baseline LVEF (%) | 42.8 (5.6) | 41.7 (6.3) | 0.8 | 60.6 (4.2) | 60.8 (6.4) | 0.8 | 60.1 (4.1) | 59.4 (5.0) | 0.7 |
| All-Cause Mortality | 5 (22%) | 3 (14%) | 0.7 | 4 (13%) | 5 (15%) | >0.9 | 9 (3.5%) | 9 (11%) | **0.019** |
| Heart Failure Hospitalization | 1 (4.3%) | 2 (9.5%) | 0.6 | 3 (9.7%) | 8 (24%) | 0.12 | 2 (0.8%) | 5 (6.3%) | **0.010** |
| Graft Failure (Censored for Death) | 1 (4.3%) | 2 (9.5%) | 0.6 | 2 (6.5%) | 4 (12%) | 0.7 | 3 (1.2%) | 6 (7.5%) | **0.007** |
| Note: All patients were included in the primary survival analysis. While 209 patients had a documented post-transplant echocardiographic encounter, the "Detailed Follow-up Echo" group (n=134) represents the subset of patients with a comprehensive examination (e.g., LVEF assessment).  Abbreviations: BMI = Body mass index; HFpEF = Heart failure with preserved ejection fraction; HFrEF = Heart failure with reduced ejection fraction; LVEF = Left ventricular ejection fraction. | | | | | | | | | |

**Supplementary Table S2**. Clinical Outcomes and Cardiovascular Interventions.

| **Variable** | **HFrEF**  (n= 44) | **HFpEF**  (n= 64) | **Control**  (n= 334) | **p-value** |
| --- | --- | --- | --- | --- |
| **Primary and Secondary Outcomes** | | | | |
| Composite (Death or HF Hosp) | 9 (20%) | 20 (31%) | 23 (6.9%) | <0.001 |
| All-Cause Mortality | 8 (18%) | 9 (14%) | 18 (5.4%) | 0.002 |
| HF Hospitalization | 3 (6.8%) | 11 (17%) | 7 (2.1%) | <0.001 |
| Graft Failure (Dialysis/Re-KT) | 3 (6.8%) | 6 (9.4%) | 9 (2.7%) | 0.022 |
| **Coronary Interventions** | | | | |
| PCI - Pre-KT | 18 (41%) | 6 (9.4%) | 19 (5.7%) | <0.001 |
| PCI - Post-KT | 4 (9.1%) | 3 (4.7%) | 13 (3.9%) | 0.2 |
| CABG - Pre-KT | 7 (16%) | 5 (7.8%) | 10 (3.0%) | 0.001 |
| CABG - Post-KT | 0 (0%) | 0 (0%) | 2 (0.6%) | >0.9 |
| Total Coronary Events – Pre-KT | 1.36 ± 1.78 | 0.45 ± 1.13 | 0.14 ± 0.50 | <0.001 |
| Total Coronary Events – Post-KT | 0.14 ± 0.41 | 0.16 ± 0.41 | 0.07 ± 0.31 | 0.2 |
| **Arrhythmia Interventions** | | | | |
| Pre-KT | 3 (6.8%) | 10 (16%) | 9 (2.7%) | <0.001 |
| Post-KT | 2 (4.5%) | 6 (9.4%) | 3 (0.9%) | <0.001 |
| **Valvular Interventions** | | | | |
| Pre-KT | 2 (4.5%) | 3 (4.7%) | 3 (0.9%) | 0.026 |
| Post-KT | 0 (0%) | 4 (6.3%) | 2 (0.6%) | 0.008 |
| Values are n (%) or Mean ± SD. P-values calculated using Chi-square test or Fisher's exact test for categorical and One-way ANOVA for continuous variables. | | | | |
| Abbreviations: KT = Kidney transplantation; HF = Heart failure; CABG = Coronary artery bypass grafting; PCI = Percutaneous coronary intervention. | | | | |
| Arrhythmia interventions include ablation, electrical/medical cardioversion, pacemaker, or ICD. Valvular interventions include TAVI/SAVR, mitral valve repair/replacement, or tricuspid intervention. | | | | |

**Supplementary Table S3**. Sequential Multivariable Cox Analysis Presenting Associations of Heart Failure Phenotypes with Clinical Outcomes Across Three Adjustment Models.

|  | **Model 0** (Unadjusted) | | | **Model 1** (Demographics) | | | **Model 2**  (Cardio-Metabolic) | | | **Model 3**  (Full Adjustment) | | |
| --- | --- | --- | --- | --- | --- | --- | --- | --- | --- | --- | --- | --- |
| **Characteristic** | **HR** | **95% CI** | **p-value** | **HR** | **95% CI** | **p-value** | **HR** | **95% CI** | **p-value** | **HR** | **95% CI** | **p-value** |
| Primary Composite (Death or HF Hospitalization) |  |  |  |  |  |  |  |  |  |  |  |  |
| Control | — | — |  | — | — |  | — | — |  | — | — |  |
| HFrEF | 3.51 | 1.62, 7.63 | **0.002** | 2.28 | 1.03, 5.02 | **0.042** | 2.82 | 1.20, 6.63 | **0.017** | 2.82 | 1.22, 6.52 | **0.016** |
| HFpEF | 5.95 | 3.24, 10.9 | **<0.001** | 5.15 | 2.76, 9.62 | **<0.001** | 4.70 | 2.50, 8.85 | **<0.001** | 4.46 | 2.36, 8.42 | **<0.001** |
| All-Cause Mortality |  |  |  |  |  |  |  |  |  |  |  |  |
| Control | — | — |  | — | — |  | — | — |  | — | — |  |
| HFrEF | 3.76 | 1.63, 8.66 | **0.002** | 2.37 | 1.01, 5.57 | **0.047** | 3.10 | 1.21, 7.95 | **0.018** | 2.90 | 1.16, 7.25 | **0.023** |
| HFpEF | 2.92 | 1.31, 6.53 | **0.009** | 2.20 | 0.96, 5.04 | 0.062 | 1.97 | 0.85, 4.59 | 0.12 | 1.68 | 0.73, 3.90 | 0.2 |
| HF Hospitalization |  |  |  |  |  |  |  |  |  |  |  |  |
| Control | — | — |  | — | — |  | — | — |  | — | — |  |
| HFrEF | 4.30 | 1.07, 17.2 | **0.039** | 2.88 | 0.71, 11.8 | 0.14 | 3.14 | 0.71, 13.9 | 0.13 | 3.11 | 0.71, 13.6 | 0.13 |
| HFpEF | 11.7 | 4.33, 31.9 | **<0.001** | 11.1 | 3.97, 30.9 | **<0.001** | 9.89 | 3.50, 28.0 | **<0.001** | 9.57 | 3.37, 27.2 | **<0.001** |
| Death-Censored Graft Survival |  |  |  |  |  |  |  |  |  |  |  |  |
| Control | — | — |  | — | — |  | — | — |  | — | — |  |
| HFrEF | 3.06 | 0.82, 11.4 | 0.095 | 2.27 | 0.59, 8.71 | 0.2 | 2.52 | 0.61, 10.5 | 0.2 | 2.39 | 0.60, 9.53 | 0.2 |
| HFpEF | 4.32 | 1.50, 12.4 | **0.007** | 3.43 | 1.16, 10.2 | **0.026** | 3.22 | 1.07, 9.71 | **0.038** | 2.60 | 0.88, 7.66 | 0.084 |
|  | | | | | | | | | | | | |
| Model 1: Adjusted for age, sex. | | | | | | | | | | | | |
| Model 2: Adjusted for age, sex, diabetes mellitus, ischemic heart disease. | | | | | | | | | | | | |
| Model 3: Adjusted for age, sex, diabetes mellitus, ischemic heart disease, and dialysis vintage.  Abbreviations: CI = Confidence interval; HFpEF = Heart failure with preserved ejection fraction; HR = Hazard ratio; HFrEF = Heart failure with reduced ejection fraction. | | | | | | | | | | | | |

**Supplementary Table S4**. Competing Risk Analysis for Graft Loss.

|  | **Unadjusted** | | | **Adjusted Model 3*** | | |
| --- | --- | --- | --- | --- | --- | --- |
| **Characteristic** | **HR** | **95% CI** | **p-value** | **HR** | **95% CI** | **p-value** |
| Graft Loss |  |  |  |  |  |  |
| Control | — | — |  | — | — |  |
| HFrEF | 2.80 | 0.75, 10.4 | 0.12 | 2.16 | 0.62, 7.49 | 0.2 |
| HFpEF | 3.88 | 1.38, 10.9 | **0.010** | 2.36 | 1.04, 5.33 | **0.040** |
| Subdistribution Hazard Ratios (sHR) for Graft Loss, Accounting for Death as a Competing Risk (Fine-Gray method). | | | | | | |
| *Adjusted model includes Age, Sex, Diabetes Mellitus, Ischemic Heart Disease, and Dialysis Vintage. | | | | | | |
